# Use of Genome Engineering to Create Patient Specific *MLL* Translocations in Primary Human Hematopoietic Stem and Progenitor Cells

**DOI:** 10.1371/journal.pone.0136644

**Published:** 2015-09-09

**Authors:** Erin H. Breese, Corina Buechele, Catherine Dawson, Michael L. Cleary, Matthew H. Porteus

**Affiliations:** 1 Division of Pediatric Hematology/Oncology, Department of Pediatrics, Stanford University, Stanford, California, United States of America; 2 Department of Pathology, Stanford University, Stanford, California, United States of America; 3 Division of Pediatric Stem Cell Transplantation and Regenerative Medicine, Department of Pediatrics, Stanford University, Stanford, California, United States of America; Emory University/Georgia Insititute of Technology, UNITED STATES

## Abstract

One of the challenging questions in cancer biology is how a normal cell transforms into a cancer cell. There is strong evidence that specific chromosomal translocations are a key element in this transformation process. Our studies focus on understanding the developmental mechanism by which a normal stem or progenitor cell transforms into leukemia. Here we used engineered nucleases to induce simultaneous specific double strand breaks in the *MLL* gene and two different known translocation partners (*AF4* and *AF9*), which resulted in specific chromosomal translocations in K562 cells as well as primary hematopoietic stem and progenitor cells (HSPCs). The initiation of a specific *MLL* translocation in a small number of HSPCs likely mimics the leukemia-initiating event that occurs in patients. In our studies, the creation of specific *MLL* translocations in CD34+ cells was not sufficient to transform cells *in vitro*. Rather, a variety of fates was observed for translocation positive cells including cell loss over time, a transient proliferative advantage followed by loss of the clone, or a persistent proliferative advantage. These studies highlight the application of genome engineering tools in primary human HSPCs to induce and prospectively study the consequences of initiating translocation events in leukemia pathogenesis.

## Introduction

Leukemia is the most common form of childhood cancer, affecting approximately 5.2 per 100,000 children per year [1]. Over the past 60 years, scientific inquiry and advancements in treatment through clinical trials have taken what used to be a uniformly fatal disease and transformed it into a disease in which more than 90% of patients are cured [2, 3]. However, certain subtypes of pediatric leukemia remain difficult to treat and retain a poor prognosis. Many of these, including the majority of cases of infant leukemia and many treatment-related leukemias, are characterized by a translocation within the *Mixed Lineage Leukemia* (*MLL*) gene [4].

Aberrations of the *MLL* gene can be found in both primary and treatment-related acute leukemia in children and adults. However, the highest frequency of *MLL* rearrangements is seen in infants with acute leukemia [5]. For infants diagnosed with acute lymphoblastic leukemia (ALL), approximately 60–80% have an *MLL* rearrangement, which has been identified as a molecular feature associated with a very poor prognosis, with overall survival less than 50% [5, 6]. For infants diagnosed with AML (acute myeloid leukemia), approximately 40% are found to have an *MLL* rearrangement [5]. While over 60 different translocation partners have been identified, the *MLL-AF4* and *MLL-AF9* translocations account for over half of the *MLL* rearrangements seen in infant leukemia [5, 6]. Interestingly, the *MLL-AF4* translocation is seen almost exclusively in ALL, while the *MLL-AF9* translocation is more commonly seen in AML, but can also occur in ALL [7]. Translocations of the *MLL* gene appear to be a driving force in the pathogenesis of leukemia in these cases, with the resulting fusion protein sustaining aberrant expression of developmental genes critical in hematopoiesis [8]. Many attempts to model this process have involved forced expression of an *MLL* fusion protein in cells using a retroviral vector [8]. While these models have advanced our understanding of the *MLL* gene and fusion proteins, they have not fully recapitulated the clinical course seen in pediatric patients [9]. We believe that a system that more accurately models the initiating events that occur in nature will provide insight into the pathogenesis and possible treatments for this disease.

Chromosomal translocations, which are a hallmark of cancer cells, have been shown to result from mis-repair of simultaneous double-strand breaks (DSBs) on two different chromosomes [10–12]. The free end of one chromosome is ligated to a portion of a different chromosome either through classic or alternative non-homologous end-joining [13]. The evidence that DSBs on two different chromosomes can cause translocations came from studies in which recognition sites for specific nucleases were introduced into two different chromosomes and then translocations between the two artificial sites measured [10]. In the last ten years, several different platforms for engineering nucleases to induce double strand breaks at specific genome target sites have been developed giving rise to the field of genome editing. These platforms include zinc finger nucleases (ZFNs), TAL effector nucleases (TALENs) and RNA-guided endonucleases of the CRISPR/Cas9 family (RGENs). These new nuclease platforms have been used to engineer translocations and chromosomal rearrangements found in Ewing sarcoma, anaplastic large cell lymphoma, and lung cancer [12, 14].

Here we designed TALENs, which consist of a fusion of a sequence specific TAL effector DNA binding domain to the nuclease domain from FokI, to specifically engineer chromosomal translocations involving the *MLL* gene in both K562 cells and primary hematopoietic stem and progenitor cells (HSPCs). We found that the frequency of translocations is higher in K562 cells than in HSPCs. Interestingly, the creation of *MLL* translocations in HSPCs was not sufficient to fully transform the cells *in vitro* into leukemia. Instead we found that there is a heterogeneous response to the creation of an *MLL* translocation whereby some cells develop a clear proliferative advantage, others develop a clear proliferative disadvantage, while still others develop a transient proliferative advantage that then disappears. These studies, which model how leukemia might occur in humans through the generation of patient specific translocations involving the endogenous genes in a small number of cells, provide the genetic foundation for studying leukemogenesis.

## Materials and Methods

### TALEN construction and validation

*MLL*, *AF4*, and *AF9* cleavage sites were designed based on patient specific translocation breakpoints available through GenBank using the TAL Effector Nucleotide Targeter 2.0 [15]. Three pairs of TALENs were created for each cleavage site using the Golden Gate TALEN Assembly Method [16] and inserted into the MR015 vector. Each pair was then tested for cutting efficiency using the surveyor assay [17]. Briefly, K562 cells were nucleofected with a combination of TALEN pairs. Genomic DNA was isolated using the Qiagen DNeasy DNA isolation system and the region of interest (*MLL*, *AF4*, or *AF9* cut sites) was amplified by PCR with the following primers: MLL_AF4_ 5’GGTTTGACCAATTGTCCCAATAAT3’, 5’TCTGGTTTGTCCTTTCCATTTGTA3’; AF4 5’TTTGGGAGACACTGGGGTAACAAT3’, 5’CCCACCTGAGGAATTTCACCTTCT3’; MLL_AF9_: 5’AGCAATCTCACAGGGTTCCT3’, 5’TGGGACAATTGGTCAAACCT3’; AF9 5’ATCTTGTTCCAGTAGAAGGCTGTTTC3’, 5’AACTCATGAATGATAAGGAAGCAAAA3’. Following isolation by gel extraction, the PCR products were heated to 95°C to denature DNA and then cooled slowly to allow the strands to re-anneal. The samples were incubated for 1h at 37°C with the T7 endonuclease, which cleaves at sites of DNA mismatches. The samples were then run on a 10% polyacrylamide gel and stained with ethidium bromide for visualization showing the expected cleaved products according to the TALENs cut sites (MLL_AF4_: ~402 bp + 68 bp; AF4: ~383 bp + 260bp; MLL_AF9_: ~240bp + 198bp; AF9: ~240bp + 112bp).

### Cell culture and nucleofection

K562 (ATCC CCL-243) cells were cultured in RPMI + 10% BGS supplemented with penicillin/streptomycin and L-glutamine and maintained at 37°C throughout the experiment. K562 cells were nucleofected using the Amaxa Nucleofector II (program T-016). Primary human CD34+ cells were isolated from fresh umbilical cord blood obtained from the maternity ward of Stanford Hospital which was approved by (Stanford University Research Compliance Office Human Subjects Research and Institutional Review Board (IRB) protocol 23665 and Stem Cell Research Oversight (SCRO) Compliance Panel protocol 401) using Ficoll-Paque plus (GE Healthcare Life Sciences) followed by Miltenyi CD34 MicroBead Kit or autoMACS cell separation. Written informed consent was obtained for use of these samples in research. They were not procured from a tissue bank or donation center. When fresh umbilical cord blood was not available, commercially available frozen human CD34+ cells isolated from umbilical cord blood were used (AllCells LLC or StemCell Technologies). Following isolation, CD34+ cells were maintained in serum free X-Vivo15 media (Lonza) supplemented with cytokines (PeproTech: SCF 100 ng/ml, TPO 100 ng/ml, Flt3L 100 ng/ml, IL-6 100 ng/ml; Cellagen Technology: SR1 0.75 μM) overnight at low oxygen tension. The following day, CD34+ cells were nucleofected using the Lonza 4-D Nucleofector System (program EO-100). Cells were incubated at 37°C, 5% CO_2_ in serum free media + cytokines + 20 μM Z-Vad-FMK (Enzo Life Sciences) to inhibit apoptosis for 48 hours following nucleofection. After 48 hours, CD34+ cell cultures were maintained in media + cytokines + 10% filtered umbilical cord blood plasma. Viability of cells was determined by flow cytometry based on *FSC and SSC* characteristics using the BD Accuri C6 flow cytometer and further confirmed by staining of Trypan blue (Life technologies) at indicated time points.

### Ethics Statement

The use of human cord blood derived CD34+ cells was approved by the Stanford University Research Compliance Office Human Subjects Research and Institutional Review Board (IRB) protocol 23665 and Stem Cell Research Oversight (SCRO) Compliance Panel protocol 401. Written informed consent was obtained for use of these samples in research. They were not procured from a tissue bank or donation center.

### Translocation PCR and Sequencing

*MLL* translocations were assayed using semi-quantitative PCR. For each PCR, 150 ng genomic DNA was used with translocation specific primers: *MLL-AF4* (*MLL* primer: 5’GGTTTGACCAATTGTCCCAATAAT3’; *AF4* primer: 5’CCCACCTGAGGAATTTCACCTTCT3’; annealing temp 60°C), *AF4-MLL* (*AF4* primer: 5’TTTGGGAGACACTGGGGTAACAAT3’; *MLL* primer: 5’TCTGGTTTGTCCTTTCCATTTGTA3’; annealing temp 59°C), *MLL-AF9* (MLL primer: 5’TGTTGACATGATTTCAGACTTACAAA3’; *AF9* primer: 5’GGAAAAGTTCTTGAATGGAATTAAAA3’; annealing temp 61°C), *AF9-MLL* (*AF9* primer: 5’AAGGCTGTTTCGTCTACATAGAAAAT3’; *MLL* primer: 5’TAGGATAATCCAGAATTTTCTTTTGC3’; annealing temp 61°C). PCR parameters: denature at 95°C for 5 min, [denature at 95°C for 30 sec, anneal at primer specific temp for 45 sec, extension at 72°C for 1 min] for 38–40 cycles depending on primer set, and final extension at 72°C for 1 min. PCR products were visualized on a 1% agarose gel with ethidium bromide. PCR products were extracted from the gel (Qiagen), cloned into the pCRII-Blunt-TOPO vector (Invitrogen), transformed into competent cells. After incubation at 37°C overnight colonies were isolated and enriched in liquid culture. Plasmid DNA of the transformed competent cells was purified (Qiagen Miniprep) and submitted for Sanger Sequencing using the M13 forward and M13 reverse primers.

### RT-PCR and qPCR

RNA was isolated from CD34+ cell cultures using the RNeasy Mini Kit (Qiagen). 500 ng of RNA from each sample was used to generate cDNA using the RevertAid First Strand DNA Synthesis Kit (Thermo Scientific). PCR was subsequently performed for the MLL-AF4 transcript [MLL primer: 5’CAGGAGGATTGTGAAGCAGAAAA3’; AF4 primer: 5’TAGGGAAAGGAAACTTGGATGG3’] using the following cycling parameters: denature at 95°C for 5 min, [denature at 95°C for 30 sec, anneal at 58°C for 45 sec, extension at 72°C for 1 min] for 36 cycles, and final extension at 72°C for 1 min. PCR products were visualized, gel extracted, and prepared for Sanger sequencing as noted above. Primers used for real-time analysis of MLL-AF4 fusion transcript were previously described by Siraj et al. [18] qPCR using Luminaris HiGreen qPCR Master Mix (Thermo Scientific) or iTaq Universal SYBR Green Supermix (BioRad) was performed using the following parameters: 95°C for 5 min, [95°C for 15 sec, 57°C for 30 sec, 72°C for 45 sec] for 40 cycles in, followed by melting curve analysis in the BioRad CFX384 C1000 Real-Time System. For real-time analysis of the genomic MLL-AF4 translocation, the MLL-AF4 primers described above were used using the following cycling parameters: 95°C for 5 min, [95°C for 30 sec, 60°C for 45 sec, 72°C for 1 min] for 39 cycles, followed by melting curve analysis. GAPDH amplification was used for normalization for all experiments. The ddCt method (Applied Biosystems) with expression levels compared relative to threshold of detection.

### Fluorescence In Situ Hybridization analysis

Live cultures of the indicated populations were submitted for FISH analysis to the Cytogenetics Laboratory of Stanford Hospital and Clinics on a fee for service basis. Cells were fixed and hybridized with the Vysis *MLL* Dual Color Break Apart Rearrangement Probe (5’ green, 3’ red). Two hundred cells were analyzed per sample for the presence or absence of an *MLL* rearrangement. A representative image was obtained for each sample.

### Colony-forming cell (CFC) assays

CD34+ cells were nucleofected with MLL and AF4 or AF9 TALENs or control (GFP) and were seeded in triplicate (10,000cells/dish) at days 25 and 39 of extended liquid culture in Methocult H4230 methylcellulose medium (StemCell Technologies Inc., Vancouver) supplemented with the same cytokines used in liquid cultures. Cultures were incubated at 37°C, 5% CO2, and colonies were scored after 12–14 days. Secondary CFC assays were performed by harvesting all cells from the primary cultures after colony enumeration, and replating them into new assays under identical conditions. Morphology of the colonies was determined as previously described [19]. All statistical analyses were performed with the Student’s *t* test. P<0.05 was considered statistically significant.

## Results

### Generation of TALENs that create specific double strand breaks within the MLL, AF4, and AF9 genes

**Fig 1:**
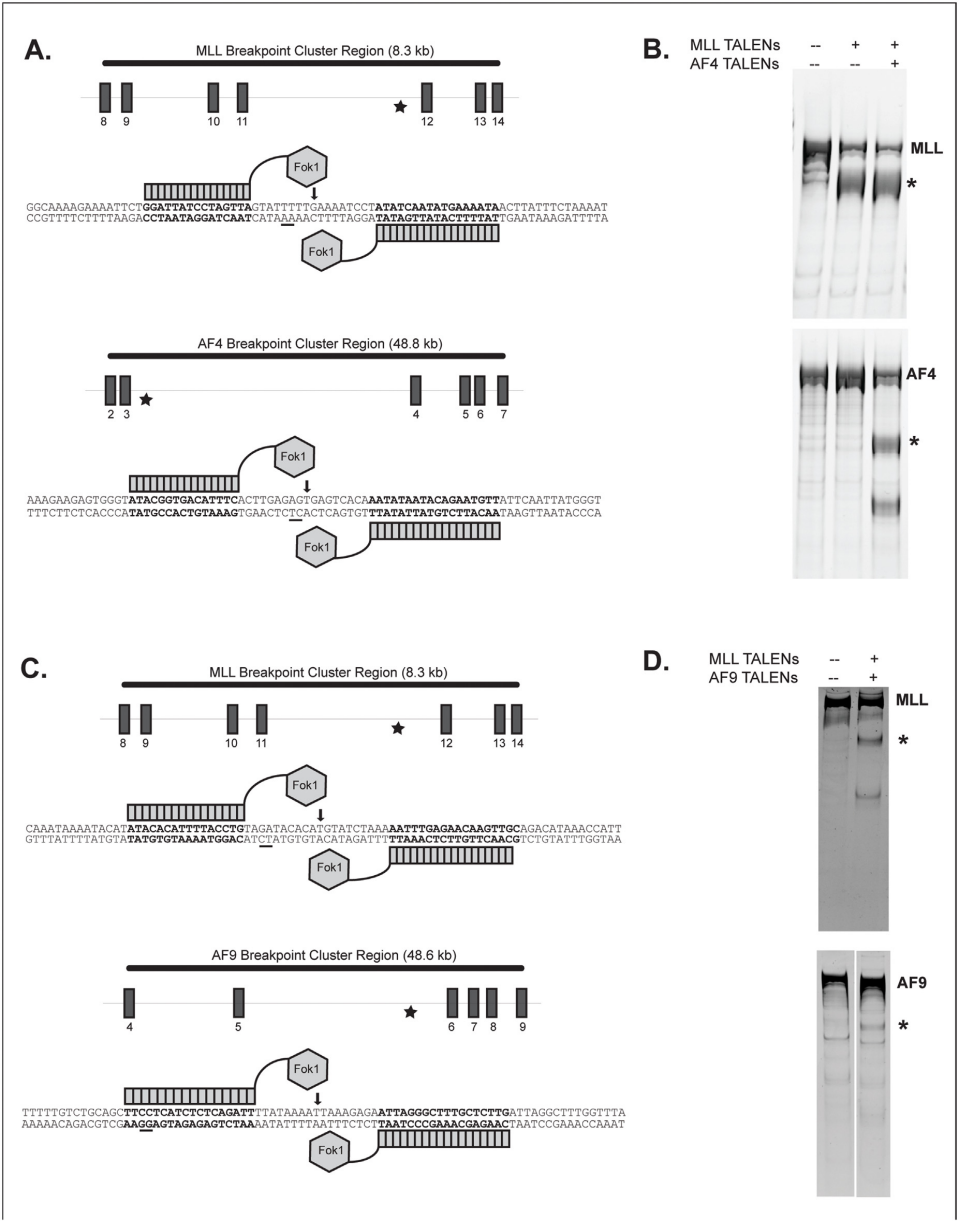
*MLL*, *AF4*, and *AF9* TALEN design. (A, C) TALEN pairs (bold sequence) were designed to target known translocation break points (underlined) within the *MLL*, *AF4*, and *AF9* genes. (B, D) Following nucleofection into K562 cells, cutting by each TALEN pair was measured using the surveyor assay, which detects indels within the target region that result from imprecise repair of a DNA double strand break. Asterisks denote cleaved PCR products.

Previous studies have identified hot spots within the *MLL*, *AF4*, and *AF9* genes, known as breakpoint cluster regions, that are frequently the sites of chromosomal translocations in patients with leukemia [7]. To design TALENs for the *MLL* and *AF4* genes, we chose a specific patient translocation sequence (UPN010) that is available through the GenBank database (AJ408902.1) and is representative of the most common site of translocations in patients with infant leukemia [20]. The Golden Gate assembly method [16] was used to create pairs of TALENs that induce a specific double strand break within the breakpoint cluster regions of *MLL* and *AF4* corresponding to the UPN010 translocation (Fig 1A). Following nucleofection with the *MLL* and *AF4* TALENs, respectively, resultant double strand breaks within the *MLL* and *AF4* genes were detected using the surveyor assay, which results in cleavage of PCR products at sites of DNA mismatch that result from the creation of indels at sites of double strand breaks (Fig 1B) [17]. A second set of TALENs was designed to create double strand breaks in the *MLL* and *AF9* genes within the breakpoint cluster regions typically seen in patients with *MLL-AF9* leukemia (Fig 1C). This design was based on the specific translocation sequence found in a child with AML (P1) that was characterized by Langer et al. [21]. The surveyor assay confirmed activity of both the *MLL* and *AF9* TALENs (Fig 1D).

### Induction of double strand breaks within the breakpoint cluster region of *MLL* and its fusion partner is sufficient to generate *MLL* translocations

**Fig 2:**
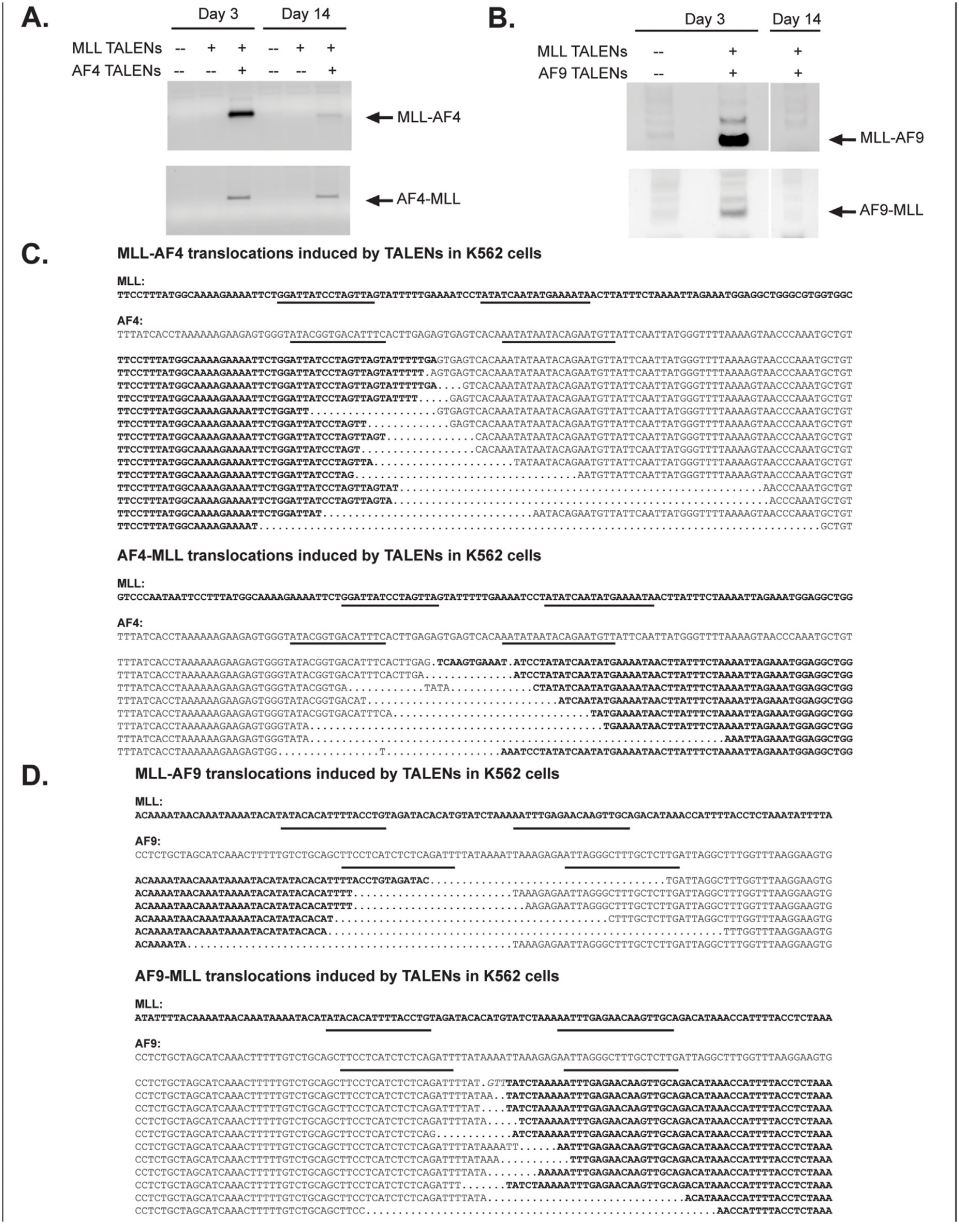
Co-expression of *MLL* and *AF4* or *AF9* TALENs in K562 cells induces *MLL* translocations. (A, B) *MLL* and *AF4* or *AF9* TALENs were transiently expressed in K562 cells. Genomic DNA was isolated at the indicated time point and used for PCR analysis for *MLL* translocations. (C, D) PCR products were isolated and sequenced to confirm translocation products. Data shown are a composite alignment of PCR products from multiple experiments showing a variety of distinct translocations. Underlines denote TALEN binding sites.

To test whether the simultaneous induction of two double strand breaks is sufficient to induce a specific chromosomal translocation, we nucleofected K562 cells with either the *MLL* and *AF4* TALENs, or the *MLL* and *AF9* TALENs. Genomic DNA isolated on day 3 post-nucleofection demonstrated both the primary *MLL* translocation (*MLL-AF4*; *MLL-AF9*) and the reciprocal translocation (*AF4-MLL*; *AF9-MLL*) (Fig 2A and 2B). Translocations were not detected when the *MLL* TALENs were expressed alone. TALEN transfected cells appeared to have a survival disadvantage in the K562 cultures as the relative intensity of the translocation product within the cell populations was significantly decreased after 14 days in culture. Resultant PCR products were isolated, and genomic rearrangements confirmed by Sanger sequencing (Fig 2C and 2D). A sub-culturing strategy was used to quantify the *MLL-AF4* translocation frequency in K562 cells, which was approximately 1 in 1100 cells (8.9 x 10^−4^). These studies demonstrate that TALENs specifically targeted to the respective breakpoint cluster regions are capable of generating *MLL* chromosomal translocations.

### TALENs targeted to breakpoint cluster regions are sufficient to generate *MLL* translocations in human primary hematopoietic stem and progenitor cells

**Fig 3:**
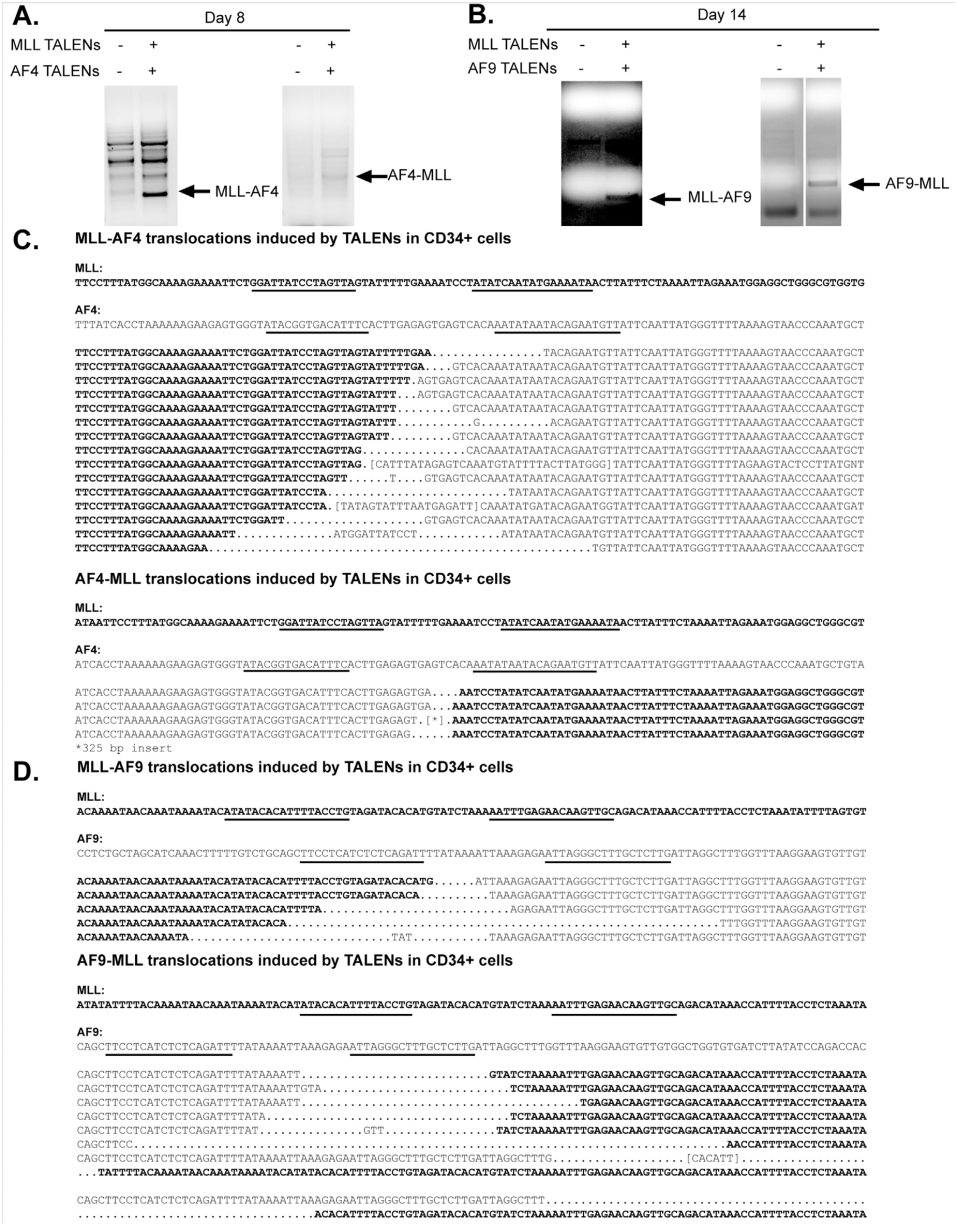
Co-expression of *MLL* and *AF4* or *AF9* TALENs in primary human CD34+ cells induces *MLL* translocations. (A, B) Primary human CD34+ cells isolated from umbilical cord blood were nucleofected with *MLL* and *AF4* or *AF9* TALENs. Genomic DNA was isolated at the indicated time point and used for PCR analysis for *MLL* translocations. (C, D) PCR products were isolated and sequenced to confirm translocation products. Nucleotide sequences shown are a composite alignment of PCR products from multiple experiments showing a variety of distinct translocations. Underlines denote TALEN binding sites.

Since K562 cells have significant chromosomal abnormalities at baseline and likely do not recapitulate what would be observed in primary cells, we explored the feasibility of inducing translocations in human primary HSPCs. After isolation of human primary CD34+ cells from fresh umbilical cord blood, the indicated TALENs were introduced by nucleofection. Cells were maintained in culture and monitored over time for the appearance of an *MLL* translocation. After 1–2 weeks following nucleofection, *MLL-AF4* or *MLL-AF9* translocations and the respective reciprocal translocations (*AF4-MLL*; *AF9-MLL*) were detected (Fig 3A and 3B). The resultant PCR products were isolated and the translocations confirmed by Sanger sequencing (Fig 3C and 3D). In order to assess the frequency of induction of *MLL-AF4* translocations by TALENs, human CD34+ cells were subdivided into a 96 well plate immediately following nucleofection with *MLL* and *AF4* TALENs (50,000 cells per well). Parallel cultures were maintained over time and monitored by PCR for the presence of the *MLL-AF4* as well as the reciprocal *AF4-MLL* translocation. The estimated *MLL-AF4* translocation frequency following nucleofection with TALENs was 2.6–4.0 x 10^−5^. The reciprocal *AF4-MLL* translocation was found to have a lower frequency of 1.3–2 x 10^−5^. Taken together, these data demonstrate the induction of *MLL* chromosomal translocations by TALEN-mediated genome engineering of primary human HSPCs.

### *MLL* translocations in primary CD34+ cells confer a survival advantage in extended culture and induce clonal expansion in colony-forming cell assays

To assess whether *de novo MLL* translocations result in a survival advantage in primary cells, human CD34+ cells were nucleofected with *MLL* and *AF4* or *AF9* TALENs or GFP and maintained in culture. Cell viability was determined by flow cytometry over time (S1 Fig). Whereas the control sample died out after 2 months in culture, the *MLL-AF4* and *MLL-AF9* samples continued to proliferate until approximately day 100–120. We did not see a difference in the proliferation capacity between the *MLL-AF4* and *MLL-AF9* samples.

**Fig 4:**
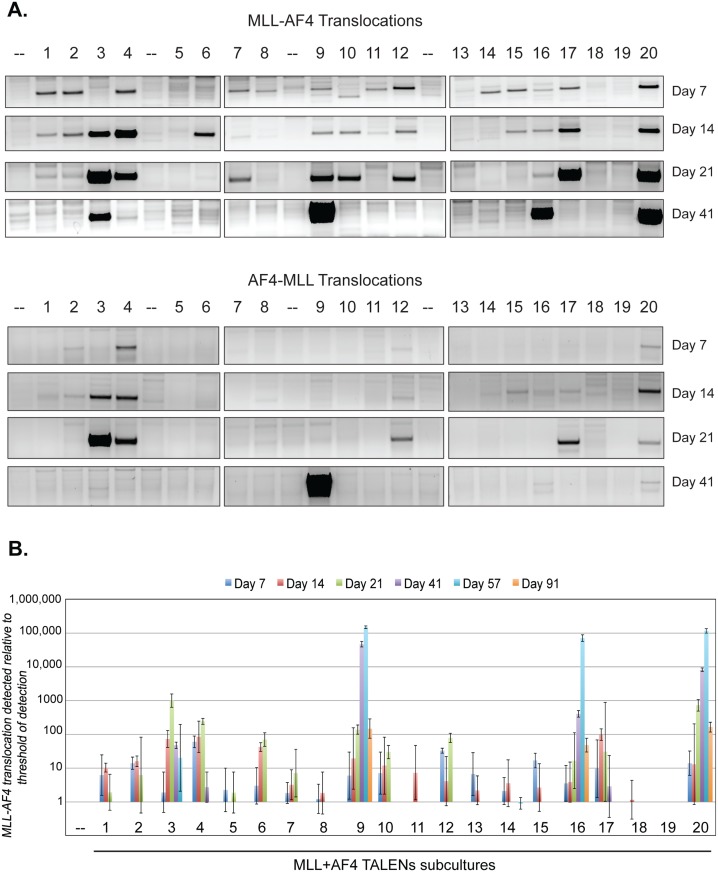
Survival advantage for CD34+ cells containing an *MLL-AF4* translocation. Primary human CD34+ cells isolated from umbilical cord blood were nucleofected with *MLL* and *AF4* TALENs. The population was immediately divided into 20 subcultures (50,000 cells per well), which were maintained over time. Genomic DNA was isolated at the indicated time points and used for semi-quantitative PCR analysis for *MLL* translocations (A) and qPCR for the MLL-AF4 translocation (B). (-- indicates subcultures nucleofected with GFP as negative control)

**Fig 5:**
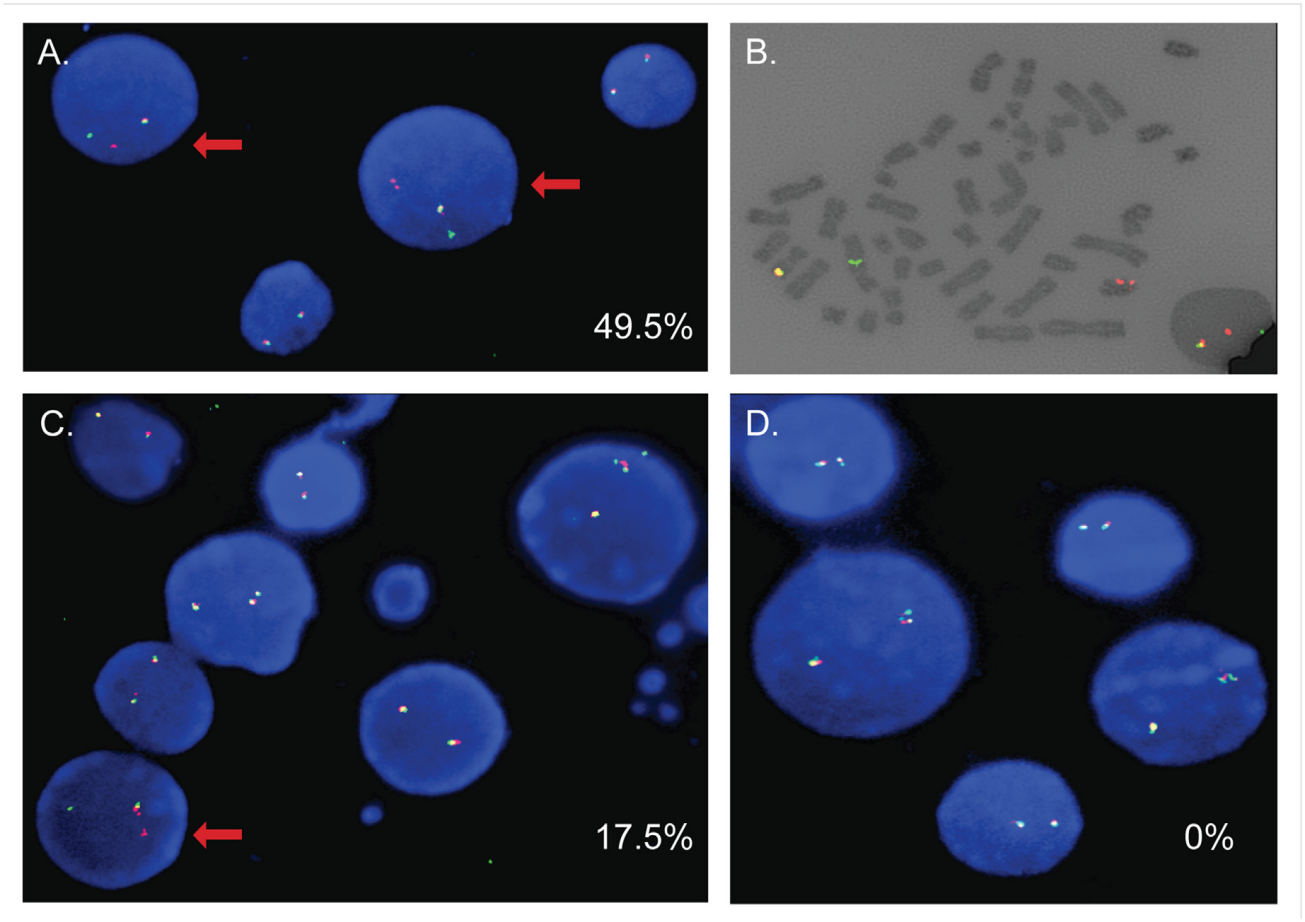
FISH analysis shows *MLL* translocations in human CD34+ cells induced by *MLL* and *AF4* TALENs. (A-D) FISH analysis using an *MLL* break apart probe was performed on cells from subcultures 9 (A, B) and 20 (C) as well as a negative control culture (D) to evaluate the presence of *MLL* chromosomal translocations. Two hundred cells were analyzed for each sample. Representative images are shown. Red arrows indicate cells positive for an *MLL* translocation. Panel B shows a cell from subculture 9 in metaphase that was noted to have an *MLL* translocation.

Further analysis of the subcultures revealed a variable pattern on a clonal level. We used both semi-quantitative PCR (*MLL-AF4* and *AF4-MLL*; Fig 4A) and qPCR (*MLL-AF4*; Fig 4B) to track the kinetics of a given clone over time. Seventeen out of the 20 subcultures had a detectable *MLL-AF4* translocation one week following nucleofection. Many of these clones persisted for 14–21 days in culture, but disappeared between days 21–41. However, a number of subcultures contained a clone with an *MLL-AF4* translocation that appeared to have a significant survival advantage as demonstrated by the increased prominence of the translocation product over time. While some of these clones displayed a rapid early expansion and subsequently disappeared after day 21, four clones remained prominent thru days 41–57. FISH analysis was performed on subcultures 9 and 20 at day 41 to better quantify the percentage of cells with an *MLL* translocation (Fig 5). Using an *MLL* break apart probe, an *MLL* translocation was detected in 49.5% (99/200) of cells in subculture 9, 17.5% (35/200) of cells in subculture 20, and 0% (0/200) of cells in the GFP control culture. Interestingly, the four subcultures that demonstrated the most significant expansion and prolonged survival in extended culture also had a detectable reciprocal *AF4-MLL* translocation by PCR (S2 Fig) and the reciprocal translocation was detected in subculture 9 by FISH analysis. Additionally, expression of the *MLL-AF4* fusion product transcript was confirmed by RT-PCR in subcultures 3, 9, 16, and 20 (S3 Fig). In order to further understand the clonal dynamics of the MLL translocations in these subcultures, *MLL-AF4* and *AF4-MLL* PCRs were repeated with genomic DNA from subcultures 3, 9, 16, and 20 and the PCR products analyzed by Sanger sequencing (S2 Fig). Sequencing confirmed 1–2 initial clones (tracked by unique translocation sequence) with one clone becoming dominant over time in each subculture.

**Fig 6:**
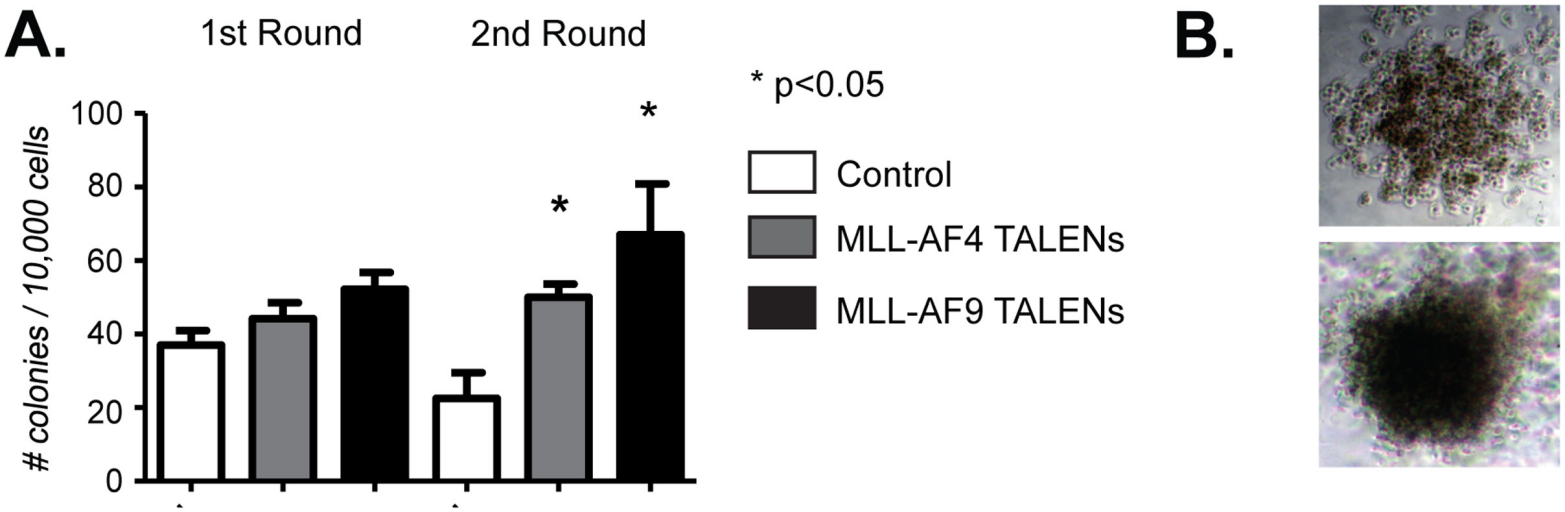
CD34+ cells nucleofected with *MLL* and *AF4* or *AF9* TALENs display enhanced replating potential in semi-solid medium. Colony forming assays were performed to assess the effect of *MLL* and *AF4* or *AF9* TALENs on the replating efficiency of CD34+ cells in semi-solid medium. The bars in Panel A represent the mean number of colonies generated per 10^4^ seeded cells. Panel B shows the morphology of secondary colonies generated by CD34+ cells nucleofected with *MLL* and *AF4* or *AF9* TALENs: diffuse colony representing ~80% (left) and compact colony representing ~20% (right) of all colonies.

We next performed colony-forming assays to further assess the clonal expansion and transformative potential of the translocated cells. For this purpose, we plated 10,000 cells of the *MLL* translocated samples (both *AF4* and *AF9*) or control samples each on day 25 and 39 of the extended culture in semisolid medium supplemented with the same cytokines that were used during the extended cultures. All three cultures generated colonies after 12–14 days, but the *MLL-AF4* and *MLL-AF9* samples displayed significantly higher clonogenic potential after the second round of replating as compared to the control samples (Fig 6A). Remarkably, approximately 20% of the colonies in the experimental samples containing the translocated cells, but not the control sample, demonstrated a compact morphology consistent with more immature cells [19] (Fig 6B). However, the colony forming assays confirmed that the translocated cells were not fully transformed since further replating resulted in decreasing cell numbers comparable to what was observed in our extended *in vitro* cultures.

## Discussion

Using tools developed for genome editing/engineering, we induced patient specific *MLL* chromosomal translocations in primary human hematopoietic stem and progenitor cells (freshly isolated CD34+ cells), and prospectively investigated what happened to the cells as a result of the initiating event. We believe that this is the first example in which TALEN nucleases have induced the relevant translocation in primary human HSPCs, a critically important milestone in using engineered nucleases to study the ontogeny of translocation associated leukemia.

*MLL*-rearranged leukemia presents a unique opportunity to explore leukemia pathogenesis in a prospective manner. We know from infant studies that *MLL* rearrangements are an initiating event that occurs *in utero* in infants who develop *MLL*-rearranged leukemia [22, 23]. Other chromosomal translocations such as *TEL-AML1* and *BCR-ABL* have been shown to occur *in utero*, but demonstrate more twin discordance, longer latency, and are thought to require subsequent mutations for leukemia development [24, 25]. Epidemiologic studies suggest that an *MLL* translocation alone may be sufficient for leukemogenesis given the high penetrance and short latency seen in twin studies [26]. *MLL* translocations also occur in therapy related leukemias in which a secondary leukemia usually occurs 1–3 years after exposure to topoisomerase inhibitors such as etoposide and teniposide [27–29]. Thus, the latency in infant leukemia and therapy related leukemia both highlight the importance of understanding the developmental period between the occurrence of the translocation and the appearance of overt leukemia.

Despite extensive investigations over the past 20 years, our understanding of the role of *MLL* translocations in the pathogenesis of acute leukemia is still not complete. Many animal models have been developed and helped to define *MLL* fusion proteins as key transcriptional and epigenetic regulators that lead to leukemia pathogenesis [7, 9, 30–34]. However, current animal models have failed to fully recapitulate the role of *MLL* fusion proteins, especially in the case of the *MLL-AF4* translocation. The *MLL-AF4* translocation has been difficult to model in mice due both to the size of the *MLL-AF4* translocation product and its chromosomal orientation [8]. Additionally, mouse models that develop leukemia by forced expression of an *MLL-AF4* fusion product have not fully recapitulated the clinical phenotype seen in infants and are complicated by potential effects related to retroviral insertion. There is ongoing debate as to whether the *MLL-AF4* fusion protein alone is sufficient to induce leukemogenesis or whether subsequent mutations are required [35]. Previous studies have demonstrated *MLL-AF4* leukemias to have fewer additional mutations as compared to other leukemias, but have identified *N-Ras*, *K-Ras*, and *Flt-3* as a source of potential cooperating mutations [36–40]. Additionally, it remains unclear whether there is a role for the reciprocal *AF4-MLL* translocation product, as only a fraction of patients have a detectable reciprocal translocation; however one report has demonstrated leukemia development in a mouse model by enforced expression of the reciprocal *AF4-MLL* fusion product alone [41]. The genome engineering approach developed in our studies provides a novel approach to more fully explore the role of various initiating events in leukemia pathogenesis in a prospective, physiologic manner using primary HSPCs.

*In vitro*, we observed that many cells with an induced *MLL* translocation do not persist in culture. This is likely a result of an intact apoptotic response in primary cells. However, a subset of cells that acquired an *MLL* translocation persisted over time, appeared to have a selective advantage in extended culture, and demonstrated a higher clonogenic potential in colony forming assays. While this effect was not as robust as that seen in previous studies when the fusion product has been introduced with retroviral vectors, this is likely related to lower expression of the fusion product under the control of the endogenous promoter. Interestingly, the clones that demonstrated the most robust survival advantage either in subculture analysis or colony forming assay contained the reciprocal translocation product. This further suggests that the reciprocal translocation may play an active role in leukemia pathogenesis. While the MLL-rearranged cells demonstrated a selective advantage, which may represent a pre-leukemic clone, our studies would suggest that they were not fully transformed as these cells eventually died out in culture as well as with serial replating in colony forming assays. This is consistent with other studies showing that enforced expression of an *MLL-AF4* transgene in primary HSPCs conveys a proliferative advantage but is insufficient to generate leukemia [9, 38]. An alternative explanation for the enhanced proliferation followed by disappearance is that the culture conditions do not support immortalized growth of transformed cells since it is widely known that primary ALL, including those with MLL translocations, are extremely difficult to grow in culture. Finally, it could also be that the clones were pre-leukemic but also died out because they retained some of the characteristics of their cell of origin (an early progenitor) and had a defined but limited replicative lifespan. Further studies are required to address these hypotheses. Nevertheless, this model system presents a unique opportunity, as we typically do not have access to pre-leukemic clones in order to explore the steps necessary to transform into leukemia. We are currently using this system both *in vitro* and *in vivo* to explore the immunophenotypic, gene expression and epigenetic changes that occur in cells over time as a result of the *MLL* translocation. Additionally, we are using this system to generate *MLL* translocations in CD34+ cells freshly isolated from umbilical cord blood followed by transplantation into immunocompromised (NSG) mice in order to develop a novel mouse model of *MLL*-rearranged leukemia. We believe this system will allow us to more fully and accurately answer whether or not *MLL* translocations are sufficient for leukemogenesis or whether they require cooperating events. It will also allow exploration of the relationship between the *MLL* translocation partner as well as cell of origin in relation to disease phenotype.

## Supporting Information

S1 Fig *MLL* translocations confer a survival advantage in primary human CD34+ cells. CD34+ cells were nucleofected with *MLL* and *AF4* or *AF9* TALENs or GFP (control) and maintained in culture *in vitro*. Cell viability was monitored over time by flow cytometry.
(TIF)

S2 Fig Clonal expansion of CD34+ cells containing an *MLL* translocation. Human CD34+ cells were nucleofected with *MLL* and *AF4* TALENs to induce *MLL* translocations. Subpopulations were sampled over time for the presence of the *MLL-AF4* and *AF4-MLL* translocations. PCR products were isolated and sequenced to evaluate evolution of sequence specific clones.
(TIF)

S3 Fig RT-PCR shows expression of *MLL-AF4* fusion transcripts in CD34+ cells induced by TALENs. (A) RNA was isolated at day 42 for each subculture showing a prominent *MLL-AF4* translocation as well as the negative control cultures. RT-PCR detected the *MLL-AF4* transcript expressed in subcultures 9 and 20. The PCR products were isolated and sequenced for confirmation. (B) Real-time RT-PCR confirmed *MLL-AF4* transcript expression in subcultures 3, 9, 16, and 20 above the threshold limit of detection.
(TIF)
