# Supplementary figures and images for: Use of Genome Engineering to Create Patient Specific MLL Translocations in Primary Human Hematopoietic Stem and Progenitor Cells
Source: PLoS One. 2015 Sep 9;10(9):e0136644. doi: 10.1371/journal.pone.0136644 (PMC4564237; doi:10.1371/journal.pone.0136644)

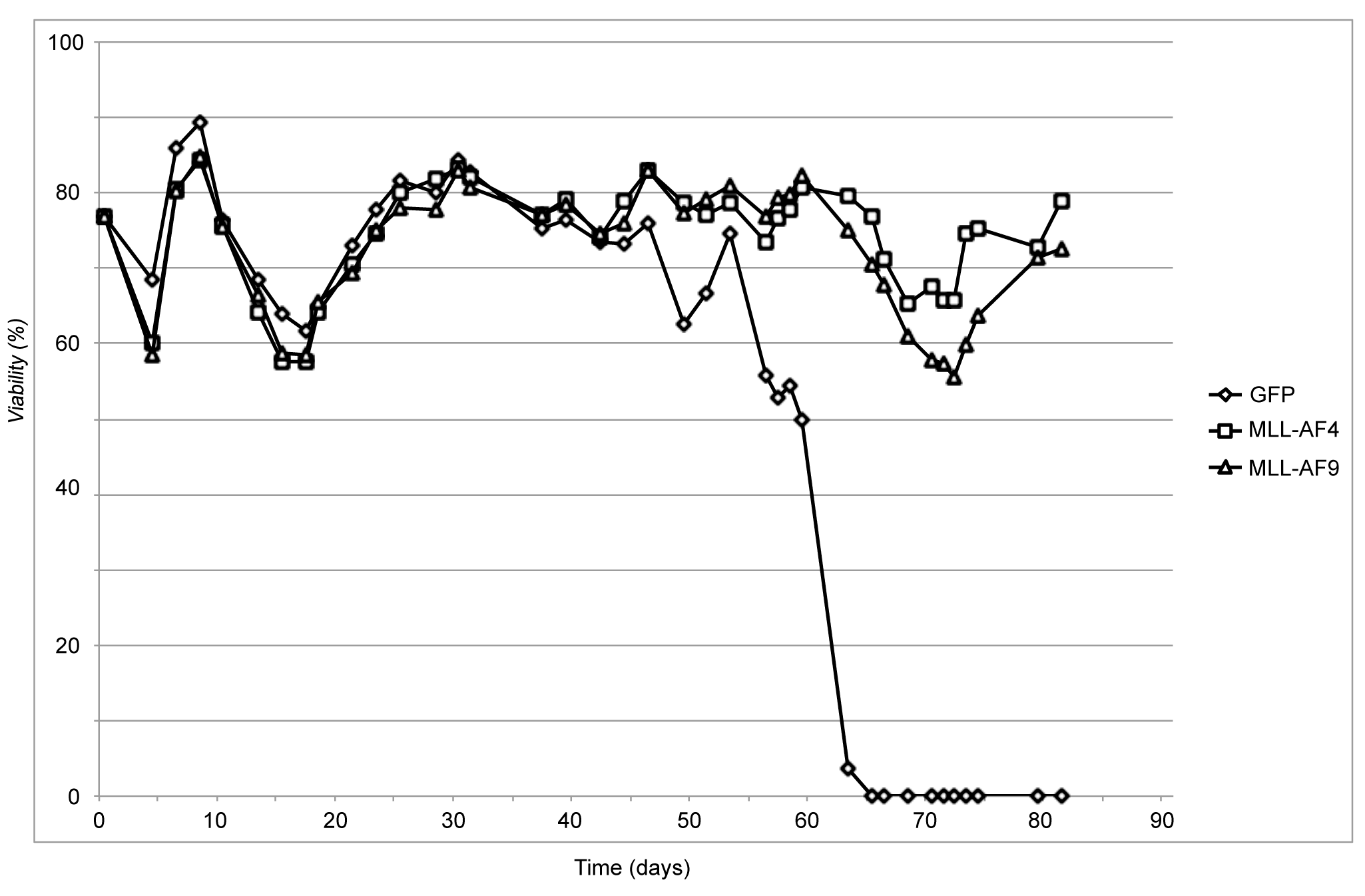

Supplement: S1 Fig — CD34+ cells were nucleofected with MLL and AF4 or AF9 TALENs or GFP (control) and maintained in culture in vitro. Cell viability was monitored over time by flow cytometry. (TIF) [file pone.0136644.s001.tif]

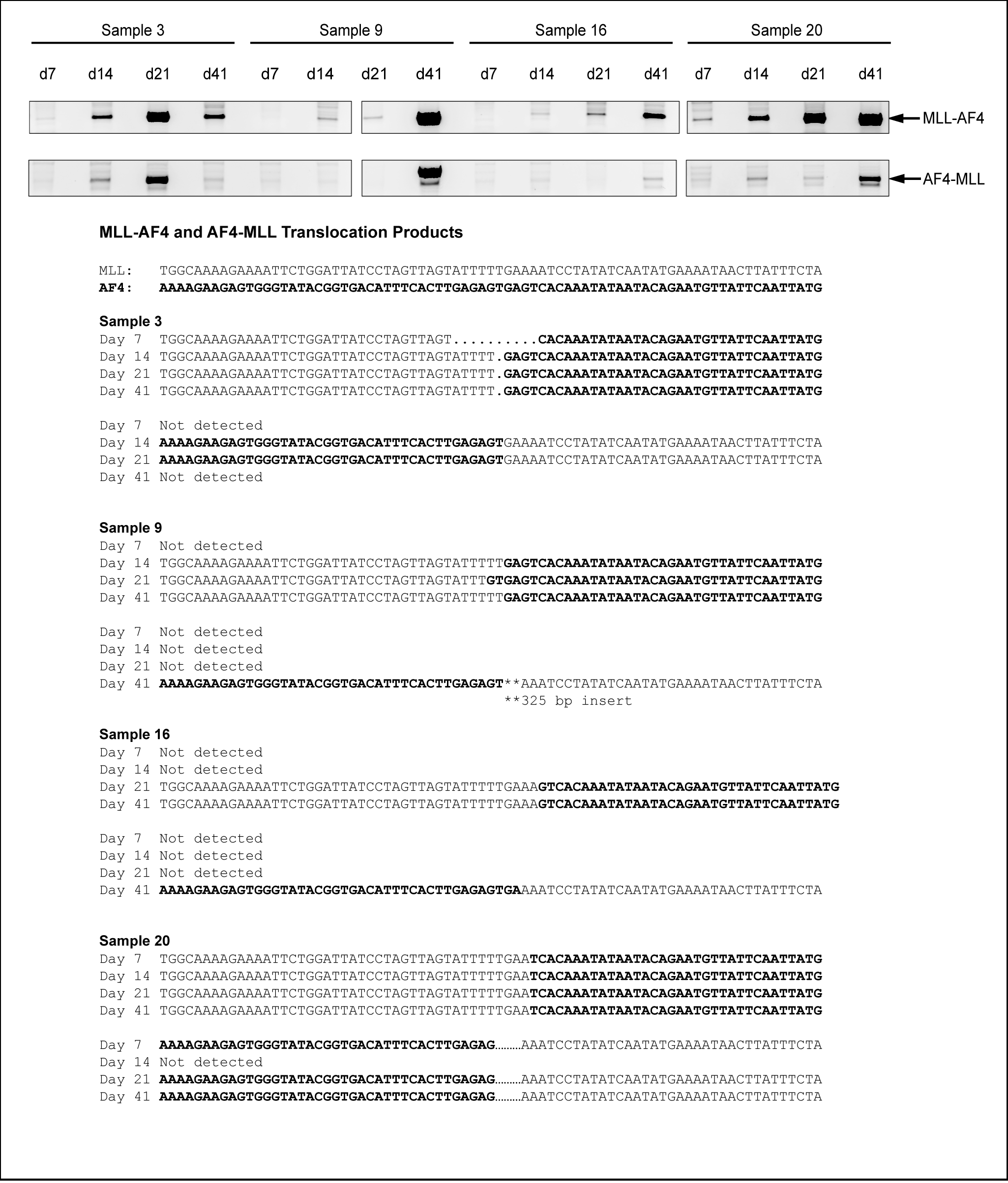

Supplement: S2 Fig — Human CD34+ cells were nucleofected with MLL and AF4 TALENs to induce MLL translocations. Subpopulations were sampled over time for the presence of the MLL-AF4 and AF4-MLL translocations. PCR products were isolated and sequenced to evaluate evolution of sequence specific clones. (TIF) [file pone.0136644.s002.tif]

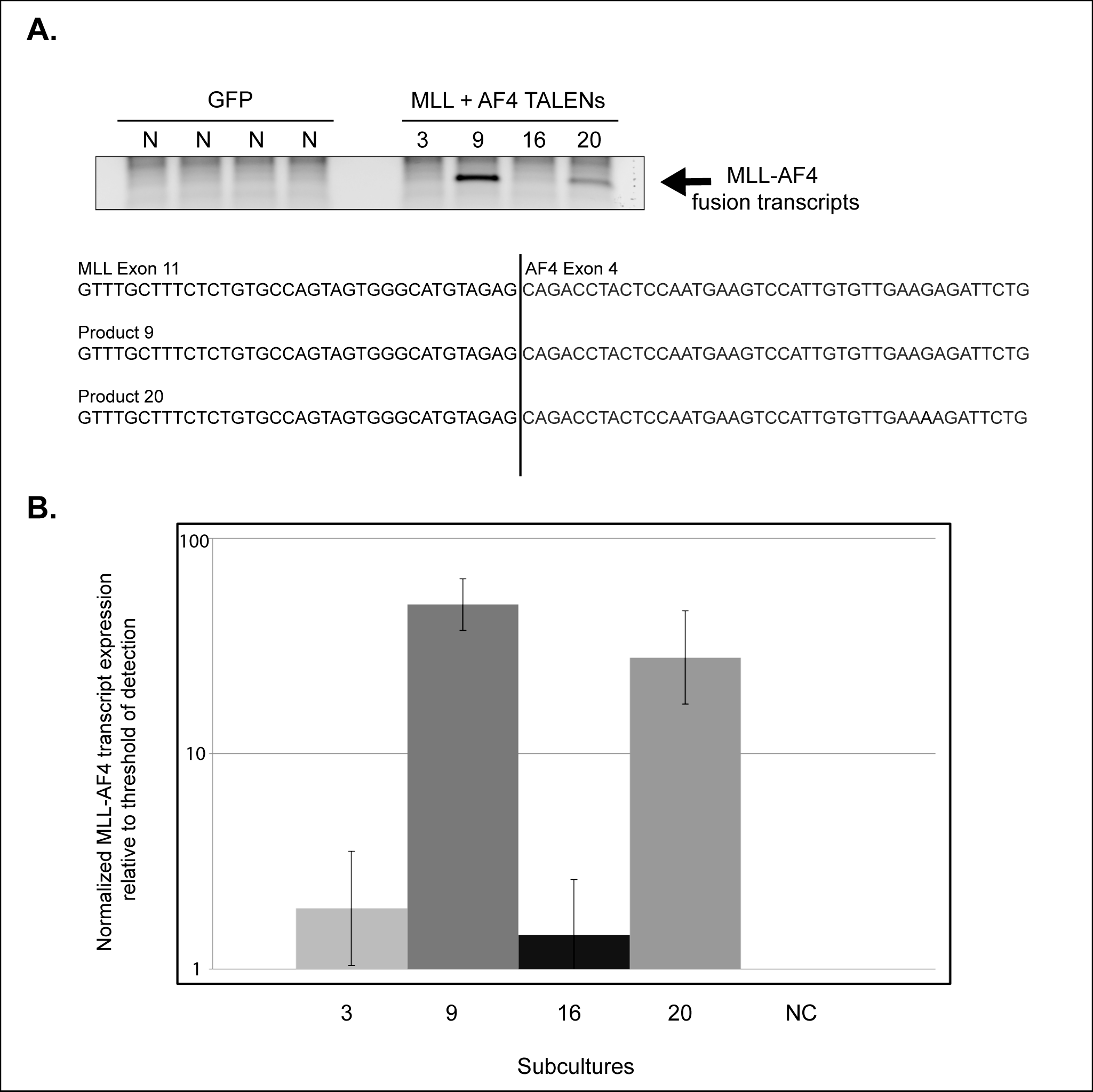

Supplement: S3 Fig — (A) RNA was isolated at day 42 for each subculture showing a prominent MLL-AF4 translocation as well as the negative control cultures. RT-PCR detected the MLL-AF4 transcript expressed in subcultures 9 and 20. The PCR products were isolated and sequenced for confirmation. (B) Real-time RT-PCR confirmed MLL-AF4 transcript expression in subcultures 3, 9, 16, and 20 above the threshold limit of detection. (TIF) [file pone.0136644.s003.tif]
